# Prevention of Incisional Hernias with Biological Mesh: A Systematic Review of the Literature

**DOI:** 10.3389/fsurg.2016.00053

**Published:** 2016-09-26

**Authors:** Filip E. Muysoms, An Jairam, Manuel López-Cano, Maciej Śmietański, Guido Woeste, Iris Kyle-Leinhase, Stavros A. Antoniou, Ferdinand Köckerling, BioMesh Study Group, Ferdinand Köckerling

**Affiliations:** ^1^Department of Surgery, Maria Middelares, Gent, Belgium; ^2^Erasmus University Medical Center, Rotterdam, Netherlands; ^3^Vall’d Hebron Hospital, Universidad Autónoma de Barcelona, Barcelona, Spain; ^4^Department of Surgery, District Hospital in Puck, Puck, Poland; ^5^Department of Radiology, Medical University of Gdansk, Gdansk, Poland; ^6^Klinikum der Johann Wolfgang Goethe-Universität, Frankfurt am Main, Germany; ^7^Center for Minimally Invasive Surgery, Hospital Neuwerk, Mönchengladbach, Germany; ^8^Department of General Surgery, University of Heraklion, Crete, Greece; ^9^Vivantes Hospital, Berlin, Germany

**Keywords:** incisional hernia, prevention, prophylaxis, biological mesh, bio-absorbable mesh, systematic review

## Abstract

**Background:**

Prophylactic mesh-augmented reinforcement during closure of abdominal wall incisions has been proposed in patients with increased risk for development of incisional hernias (IHs). As part of the BioMesh consensus project, a systematic literature review has been performed to detect those studies where MAR was performed with a non-permanent absorbable mesh (biological or biosynthetic).

**Methods:**

A computerized search was performed within 12 databases (Embase, Medline, Web-of-Science, Scopus, Cochrane, CINAHL, Pubmed publisher, Lilacs, Scielo, ScienceDirect, ProQuest, Google Scholar) with appropriate search terms. Qualitative evaluation was performed using the MINORS score for cohort studies and the Jadad score for randomized clinical trials (RCTs).

**Results:**

For midline laparotomy incisions and stoma reversal wounds, two RCTs, two case–control studies, and two case series were identified. The studies were very heterogeneous in terms of mesh configuration (cross linked versus non-cross linked), mesh position (intraperitoneal versus retro-muscular versus onlay), surgical indication (gastric bypass versus aortic aneurysm), outcome results (effective versus non-effective). After qualitative assessment, we have to conclude that the level of evidence on the efficacy and safety of biological meshes for prevention of IHs is *very low*. No comparative studies were found comparing biological mesh with synthetic non-absorbable meshes for the prevention of IHs.

**Conclusion:**

There is no evidence supporting the use of non-permanent absorbable mesh (biological or biosynthetic) for prevention of IHs when closing a laparotomy in high-risk patients or in stoma reversal wounds. There is no evidence that a non-permanent absorbable mesh should be preferred to synthetic non-absorbable mesh, both in clean or clean-contaminated surgery.

## Introduction

Prophylactic mesh-augmented reinforcement during closure of abdominal wall incisions has been proposed in patients at high risk for incisional hernia (IH). Several randomized clinical trials (RCTs) have been published on the use of prophylactic mesh in patients undergoing aortic aneurysm surgery (1–4), obesity surgery (3, 5–7), stoma creation (8–14), in colorectal cancer patients (15, 16), or other high-risk patients (17, 18). The recently published guidelines of the European Hernia Society have provided the following *weak* recommendation: *“Prophylactic mesh augmentation for an elective midline laparotomy in high-risk patients in order to reduce the risk of incisional hernias is suggested.”* Due to the lack of sufficient data, no recommendations on the type of mesh, the optimal mesh position, or the optimal mesh fixation technique could be made (19). Although prophylactic mesh-augmented reinforcement has been performed safely in clean-contaminated setting, one concern is the potential short- or long-term harms by implantation of a permanent mesh (20). Application of a non-permanent absorbable for prophylactic mesh-augmented reinforcement might therefore hold some benefit if these meshes will be as effective as permanent meshes.

A systematic literature review has been performed to detect those studies where prophylactic mesh-augmented reinforcement was performed with a non-permanent absorbable biological or biosynthetic mesh and provide guidance for future research on the use of biological or biosynthetic meshes.

## Methods

### Protocol

The systematic search was part of the BioMesh consensus project. This project, initiated by Ferdinand Köckerling, gathered surgical expertise in a working group to provide a summary on the use of non-permanent absorbable biological or biosynthetic meshes in different indications. During a consensus meeting in Berlin on January 27, 2016, the working group decided in consensus on the statements and conclusions derived from the level of evidence for each indication. This manuscript reports on the review of the use of non-permanent absorbable biological or biosynthetic meshes for the prevention of IHs.

### Eligibility Criteria

Inclusion criteria: because of the paucity of available studies on prophylactic mesh-augmented reinforcement with biological or biosynthetic mesh for the prevention of IHs, no limitation, to the study design, length of follow-up, or number of included patients, was used.

Exclusion criteria: prevention of parastomal hernias were excluded because this was part of a separate search within the BioMesh study group (21).

### Information Sources

A computerized search was performed within 12 databases (Embase, Medline, Web-of-Science, Scopus, Cochrane, CINAHL, Pubmed publisher, Lilacs, Scielo, ScienceDirect, ProQuest, Google Scholar) on June 25, 2015.

### Search

The biomedical librarian of the Erasmus University Medical Centre, Rotterdam, The Netherlands performed the search, and the search strategy is provided in Section “Addendum 1” in Appendix.

### Study Selection

From the search, only the studies reporting on the use of a non-permanent absorbable biological or biosynthetic mesh were retained. Studies written in English, Dutch, French, and Spanish were considered.

### Data Collection Process

Two authors (Filip Etienne Muysoms and An Jairam) independently screened all records retrieved upon application of the search strategy by title and abstract. The full text of all retained records was screened for eligibility. The references of all review articles found were cross-checked for additional eligible records.

### Data Items

The following data were extracted by two authors independently and cross-checked: type of study, number of patients included, patient characteristics, indication for surgery, type of biological mesh, position of the mesh, method of mesh fixation, length of follow-up, and outcome measures (hernias, seroma, wound infections, burst abdomen). Primary outcome was IH incidence, and secondary outcomes were postoperative seroma, wound infection, and burst abdomen.

### Quality Assessment of Individual Studies

Qualitative evaluation was performed using the MINORS score for non-randomized studies (22) and the Jadad score for RCTs (23). Additionally, the quality of evidence across the RCTs was done using the GRADE Pro software.^1^

### Statistical Analysis

A meta-analysis of the outcome from the RCTs detected was performed for relevant outcomes: IH, seroma, wound infections, and burst abdomen. Meta-analysis was performed using the Review Manager 5.3 software (Copenhagen: The Nordic Cochrane Centre, The Cochrane Collaboration, 2013). Our outcomes were expressed as risk ratios (RRs) with 95% confidence intervals (CIs) to estimate the pooled effect size and *p*-value. All tests were two-sided.

## Results

### Study Selection

**Figure 1:**
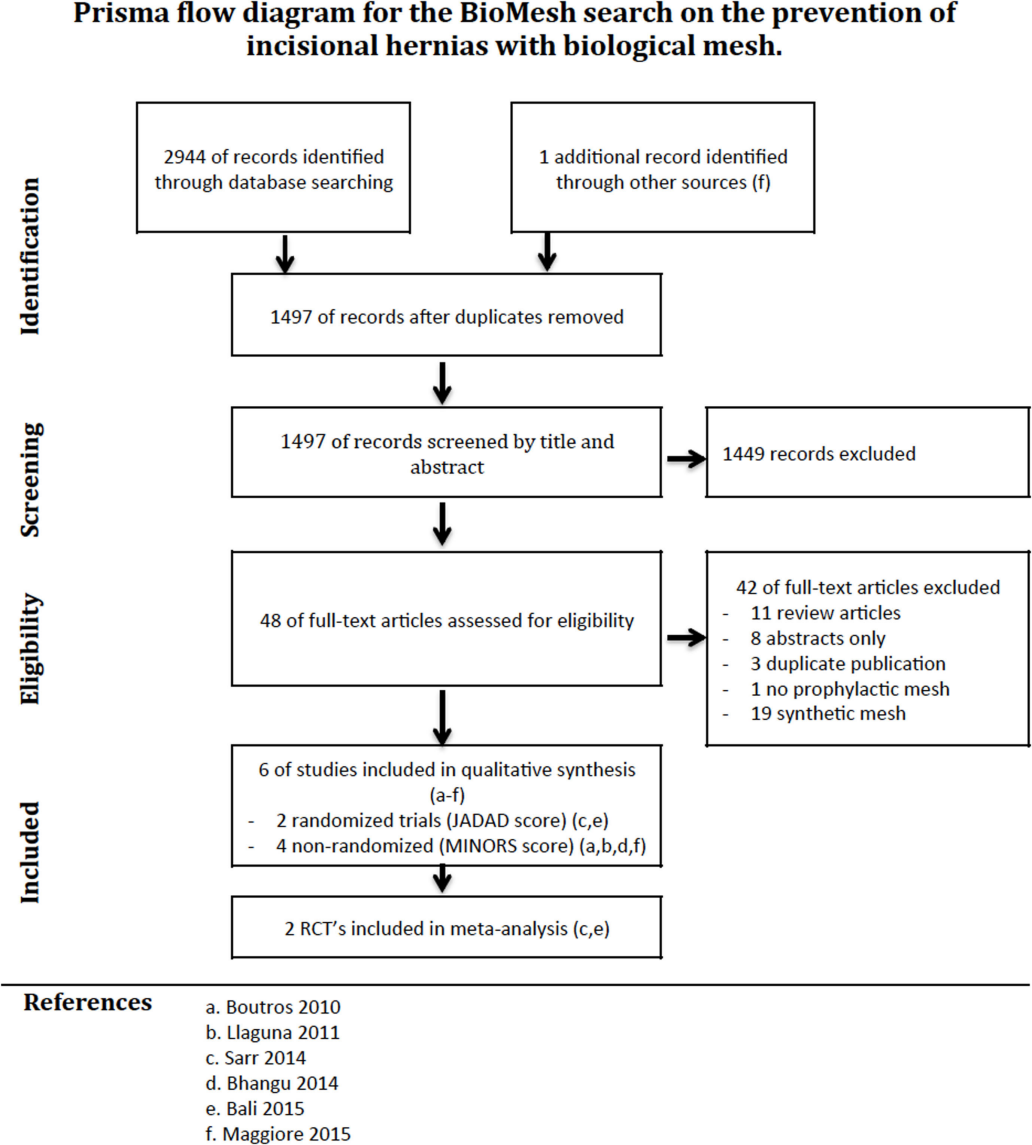
**PRISMA flow diagram of a systematic review on the use of biological mesh for prevention of incisional hernias**.

The PRISMA flow diagram of our search is illustrated in Figure 1. Six studies were retained after the screening and sift for eligibility. Four studies included patients with *midline laparotomy* (2, 7, 24, 25), and two studies investigated the prevention of IHs after *stoma reversal* (26, 27).

### Study Characteristics

#### Midline Laparotomy

Our literature review revealed four studies where a biological mesh was used to prevent IHs in high-risk patients. Details of the study characteristics and quality assessment (MINORS score, Jadad score) are shown in the summary of evidence table (Table 1). A small cohort study on eight patients that underwent a midline laparotomy for cytoreductive surgery and hyperthermic intraperitoneal chemotherapy (HIPEC) described short-term outcome using an intraperitoneal biological mesh (24). In a prospective non-randomized case–control study, obese patients operated for a gastric bypass through a midline laparotomy were either treated with an intraperitoneal biological mesh (*n* = 59) or primary suture closure (*n* = 75). A significant reduction in the number of IHs by prophylactic mesh was reported [2.3% (90% CI: 2.31–6.86) versus 17.7% (90% CI: 7.92–27.52), *p* = 0.014] (25). In an RCT in obese patients undergoing a gastric bypass operation through a midline laparotomy, patients were randomized between an intraperitoneal biological mesh (*n* = 185) and primary suture closure (*n* = 195). This adequately powered RCT, did not show any benefit for prophylactic mesh concerning the risk for IH at 24 months (17.3 versus 19.5%, *p* = 0.60), but did show a significant higher number of wound infections and wound seroma in the mesh group (7). In an RCT of aortic aneurysm patients, midline laparotomy closure with an onlay biologic mesh (*n* = 20) was compared to primary suture closure (*n* = 20) (2). The study was not powered with a sample size calculation, but the follow up was adequate in length (36 months) and methodology (systematic CT scan evaluation). A highly significant protective effect of the mesh was shown, with no hernias in the mesh group and 32% in the non-mesh group [cumulative freedom of IH at 36 months was 100 versus 74.4% (*p* < 0.008)] (2).

**Table 1:** **Summary of evidence table of a systematic review on the use of biological mesh for the prevention of incisional hernias after midline laparotomy**.

Reference	Study type	Quality assessment	*N* (mesh/no mesh)	Patient characteristics	Intervention	Comparison	Length of follow-up (months)	Outcome measure
Boutros et al. (24)	Non-comparative case series	MINORS score 5/16	8/–	Midline laparotomy for cytoreductive surgery and HIPEC in peritoneal carcinoma patients	Intraperitoneal Surgisis 20 cm × 20 cm fixed with PDS sutures	–	Mean 6.3	Seven patients had no abdominal wall morbidity. One patient had an incisional hernia and entero-cutaneous fistula following re-laparotomy 2 weeks after the primary operation

General comments: very low MINORS score of this case series. Follow-up inadequate to make conclusion about incisional hernia rate								
Funding: no direct funding; speakers fee from Cook								
Study registration: no								

Llaguna et al. (25)	Prospective case–control study	MINORS score 19/24	134 (59/75)	Patients undergoing gastric bypass surgery with midline laparotomy	Intraperitoneal Alloderm 16-cm long and 6-cm wide, fixed with PDS sutures	Sutured with PDS no 1, running suture	Mean 17.3	Incisional hernia: mesh: 1/44 (2%); no mesh: 11/62 (18%); *p* = 0.014 (OR 0.06)

General comments: prospective single surgeon non-randomized study, with adequate follow-up. Statistical significant differences on the number of patients with some confounding factors were seen: prior abdominal surgery, postoperative BMI								
Funding: not mentioned								
Study registration: no								

Sarr et al. (7)	RCT	JADAD score 2/5	402 (185/195)	Patients undergoing gastric bypass surgery with midline laparotomy	Intraperitoneal Surgisis 8-cm wide fixed with PDS sutures	Suture non-absorbable and absorbable, running suture	24	Incisional hernia: mesh: 32/185 (17.3%); no mesh: 38/195 (19.5%); *p* = 0.60; wound infections: 11.9% versus 3.6% (*p* < 0.003); wound seroma: 4.9% versus 0.5% (*p* < 0.01)

General comments: open label RCT with adequate sample calculation and power. Showed no difference in incisional hernia rate. The number of clinically relevant wound infections and wound seroma was significant higher in the Mesh group								
Funding: industry-funded study (Cook Biotech, Inc., West Lafayette, IN, USA)								
Study registration: www.ClinicalTrials.gov NCT00274625								

Bali et al. (2)	RCT	JADAD score 1/5	40 (20/20)	Elective midline laparotomy for AAA repair	Onlay periguard 8-cm wide fixed with non-absorbable sutures	Sutured with PDS no 1, running suture	36	Incisional hernia: mesh: 0/20 (0%); no mesh: 6/20 (32%); estimate freedom of incisional hernia was significantly higher for the mesh group (*p* < 0.008)

General comments: small open label RCT, no sample size calculation. Prophylactic mesh was effective and safe								
Funding: not mentioned								
Study registration: no								

#### Stoma Reversal Wound

Our literature review revealed two studies in which a biological mesh was used to prevent IHs after reversal of a temporary ileostomy. Details of the studies are shown in the summary of evidence table (Table 2). In a pilot study with a limited patient population (*n* = 7), the feasibility of an intraperitoneal prophylactic mesh was investigated in terms of safety in the short term (27). The second report was a matched case–control study of 30 patients that received a retro-muscular prophylactic biological mesh, compared to 64 matched patients with suture closure of the stoma wound. At 1-year follow-up with CT scan, the number of patients with IH was significantly lower for the mesh group (*p* = 0.043).

**Table 2:** **Summary of evidence table of a systematic review on the use of biological mesh for prevention of incisional hernias after stoma reversal**.

Reference	Study type	Quality assessment	*N (*mesh*/*no mesh)	Patient characteristics	Intervention	Comparison	Length of follow-up	Outcome measure
Bhangu et al. (26)	Non-comparative case series	MINORS score 4/16	7/–	Patients with a temporary ileostomy needing stoma closure	Intraperitoneal Strattice 3-cm overlap fixed with PDS sutures	–	30 days	One superficial wound infection. No early hernias

General comments: very low MINORS score of this case series. Follow-up inadequate to make conclusion about incisional hernia rate. This study is a pilot study on the safety of the technique, before starting a large RCT								
Funding: industry-funded study								
Study registration: part of the ROCCS study: www.ClinicalTrials.gov NCT02238964								

Maggiori et al. (27)	Matched case–control study	MINORS score 15/24	94 (30/64)	Closure of a diverting ileostomy following rectal cancer resection	Retro-muscular Meccellis mesh 10 cm × 10 cm, fixed with prolene sutures	Two layer continuous suture of anterior and posterior fascia with Vicryl 1	1 year	Radiological incisional hernia rate mesh: 1/30 (3%); no mesh: 12/64 (19%) *p* = 0.043

General comments: Significant reduction of the number of incisional hernias at the stoma wound diagnosed with CT scan. No difference in morbidity								
Funding: industry-funded study								
Study registration: no								

### Meta-analysis

**Figure 2:**
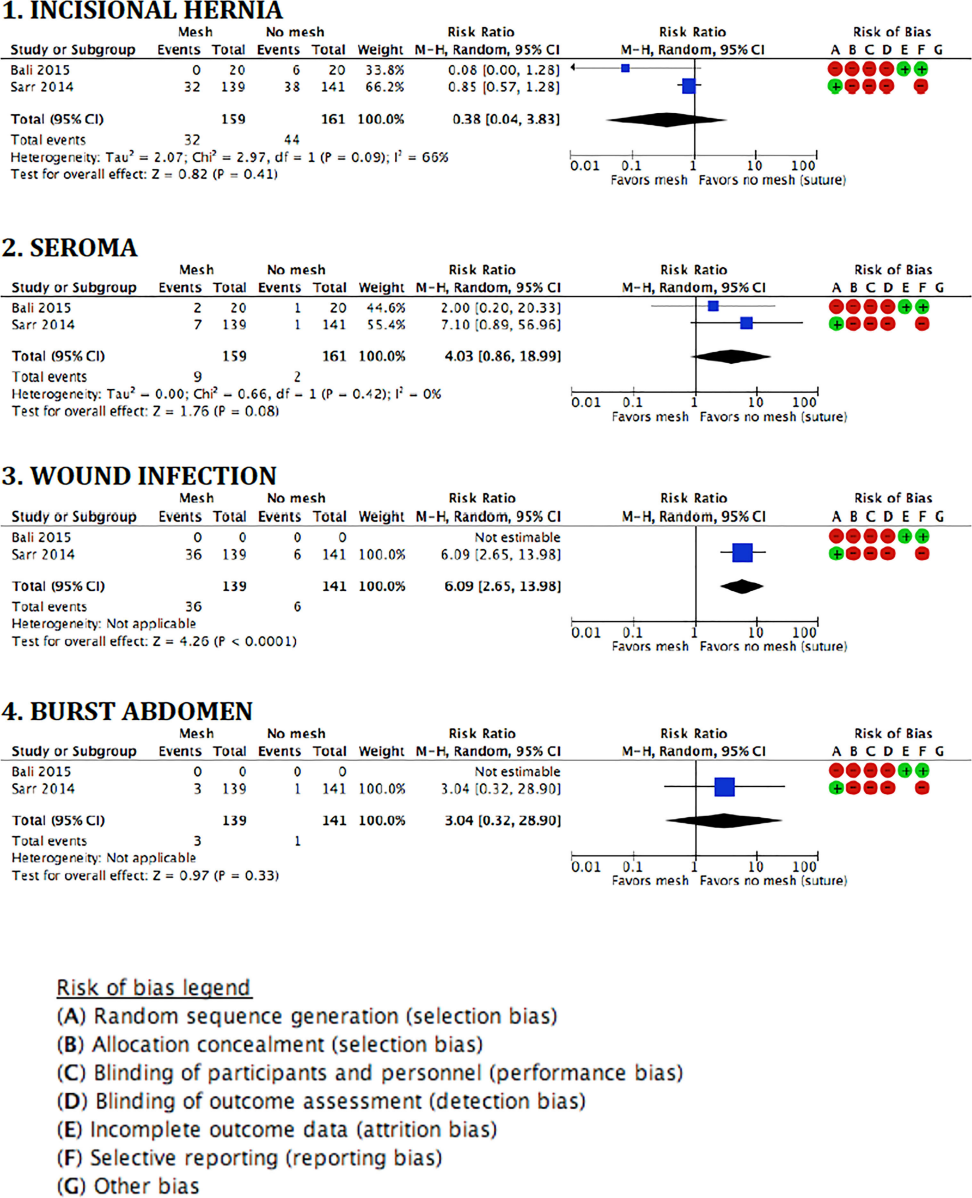
**Forest plots and risk of bias assessment of randomized studies on the prevention of incisional hernias by biological mesh reinforcement**.

The pooled analysis for the outcome IH showed no statistical differences between groups (RR 0.38, 95% CI 0.04–3.83; *p* = 0.41). The forest plots of the meta-analysis of the two RCTs on prevention of midline laparotomy IHs, and the secondary outcomes are shown in Figure 2.

## Discussion

### Midline Laparotomy

Overall, the *Level of Evidence* on the efficacy of biological mesh to prevent IHs is *very low*. Moreover, the study with the highest level of evidence and lowest risk of bias did not show any advantage in reducing IHs by prophylactic intraperitoneal biological mesh in patients undergoing a midline laparotomy for performing gastric bypass surgery (7). On the contrary, it did show a higher number of wound complications after the use of the prophylactic mesh. Another study regarding gastric bypass patients did show a benefit, but this study was non-randomized and had a high risk of bias (25).

For aortic aneurysm patients, only one RCT is available, which showed a high efficacy with 3 years follow-up. However, this study was poorly powered, non-blinded, and scored low in the Jadad scale (2). Moreover, no information on sources of funding and protocol registration was provided, and therefore, the risk of bias cannot be assessed.

The currently available evidence is not strong enough to make any statements regarding the optimal mesh position (intraperitoneal, retro-muscular, or onlay) in case a prophylactic biological mesh is used. Also, the different meshes used in the studies (non-cross-linked human origin; non-cross-linked porcine small intestinal submucosa; cross-linked bovine pericardium) might have an important impact on the outcome.

On the contrary, the Level of Evidence on the efficacy of prophylactic synthetic non-absorbable mesh (all polypropylene) in high-risk patients currently is high, with 8 published RCTs encompassing 727 patients with a follow-up of at least 12 months (1, 4–6, 15–18). Moreover, the safety of prophylactic retro-muscular or onlay meshes in clean or clean-contaminated surgery is shown in 9 published RCTs encompassing 1207 patients (1, 3–6, 15–18).

No comparative studies were found comparing biological mesh with synthetic non-absorbable meshes for the prevention of IHs. There is a study ongoing at the Vall d’Hebron Hospital, Universidad Autónoma de Barcelona on the prevention of IHs from midline laparotomies using an absorbable synthetic mesh (Bio-A, WL Gore & Ass, USA), PREBIOUS trial.^2^

### Stoma Reversal Wound

Overall, the *Level of Evidence* on the efficacy of biological mesh to prevent IHs of stoma reversal wounds is *very low*. Currently, the only study providing evidence is a matched case–control study, showing a lower IH rate at 1 year. This study is a pilot study for an RCT that is planned in France, the MEMBO trial^3^ (27). The small pilot study by Banghu et al. is part of a large project, the ROCSS study, which is a properly powered multicenter RCT from the University of Birmingham^4^ (26). This study compares the technique described in the pilot study with sutured closure of the stoma wound and has now included 790 patients, and the follow-up is ongoing. Furthermore, a study from the Vall d’Hebron Hospital (Universidad Autónoma de Barcelona), ILEOCLOSE study,^5^ will investigate in a RCT the application of prophylactic mesh reinforcement of closure of temporary diverting ileostomy with an absorbable synthetic mesh (Bio-A) in 120 patients.

## Conclusion

So far, there is no solid evidence on the effectiveness of prophylactic non-permanent absorbable biological or biosynthetic mesh for the closure of midline laparotomies or reinforcement of a stoma reversal site. There is no evidence that, in this setting, a non-permanent absorbable biological or biosynthetic mesh should be preferred to synthetic non-absorbable mesh, both in clean or clean-contaminated surgery.

## Publication Statement

This manuscript was written in accordance with the PRISMA statement: The PRISMA statement for reporting systematic reviews and meta-analyses of studies that evaluate health-care interventions: guidelines for reporting parallel group randomized trials.^6^

## Author Contributions

All authors: initiation of the project, determination of search strategy, reviewing manuscript, and agreement. FM and AJ: systematic search at Erasmus University Rotterdam, the Netherlands, data collection process, and writing the manuscript. FM, AJ, FK, and IK-L: study selection from retrieved records. FM, AJ, ML-C, SA, MS, GW, and FK: qualitative evaluation of the retrieved records. FM, AJ, ML-C, and IK-L: quantitative evaluation of the retrieved records.

## BioMesh Study Group

Ferdinand Köckerling (Chairman), Stavros Antoniou, René Fortelny, Frank A. Granderath, Markus Heiss, Franz Mayer, Marc Miserez, Agneta Montgomery, Salvador Morales-Conde, Filip Muysoms, Alexander Petter-Puchner, Rudolph Pointner, Neil Smart, Marciej Smietanski, and Bernd Stechemesser.

## Conflict of Interest Statement

Dr. FM reports grants and personal fees from Covidien, grants and personal fees from Johnson & Johnson, personal fees from WL Gore & Ass, personal fees from Bard Davol, grants and non-financial support from FEG DynaMesh, grants and personal fees from B. Braun, outside the submitted work; Dr. AJ reports no conflict of interest concerning the work. Dr. ML-C has received honoraria for consultancy and lectures from Ethicon, Johnson & Johnson, personal fees from Bard Davol, and grants from WL Gore & Ass, outside the submitted work. Dr. IK-L reports no conflict of interest concerning the work. Dr. MS reports fees for teaching and learning from Medtronic, Bard Davol, B. Braun, and Ehticon, outside of the submitted work. Dr. GW reports grants and consulting fees from Acelity, B. Braun, and Bard Davol, outside the submitted work. Dr. SA reports absence of any commercial or financial relationships that could be construed as a potential conflict of interest. Dr. FK reports no conflict of interest concerning the work.

## Footnotes

^1^www.gradepro.org

^2^www.ClinicalTrials.gov NCT02208557

^3^www.ClinicalTrials.gov NCT02576184

^4^www.ClinicalTrials.gov NCT02238964 and www.controlled-trials.com ISRCTN46330337

^5^www.ClinicalTrials.gov NCT02226887

^6^www.prisma-statement.org

## Appendix

### Addendum 1

Search strategy used for a systematic literature review on prevention of incisional hernias with mesh. A computerized search was performed within 12 databases (Embase, Medline, Web-of-Science, Scopus, Cochrane, Cinahl, Pubmed publisher, Lilacs, Scielo, ScienceDirect, ProQuest, Google scholar) on June 25th 2015.

### Embase.com 839

('surgical mesh'/exp OR (mesh* OR 4DDOME OR AIGISRx OR AlloDerm OR AlloMax OR 'Bard Composix EX' OR 'BIO-A Tissue Reinforcement prosthesis' OR CollaMend OR DermaMatrix OR DualMesh OR 'Evolution P3EM' OR FasLata OR FlexHD OR FortaGen OR 'IntePro Lite' OR InteXen OR NEOVEIL OR 'Parietex composite' OR Pelvicol OR Pelvisoft OR Pelvitex OR PerFix OR 'Peri-Strips Dry' OR PeriGuard OR Permacol OR Physiomesh OR SeamGuard OR Strattice OR Surgisis OR 'TiLoop Bra' OR Timesh OR Tutomesh OR Tutopatch OR Ultrapro OR Ventralex OR Veritas OR Vivosorb OR Vypro OR X-Repair OR XenMatrix):ab,ti) AND (prevention/exp OR prevention:lnk OR (prevent* OR protect* OR prophyla*):ab,ti) AND ('incisional hernia'/exp OR 'abdominal wall hernia'/de OR 'abdominal wall defect'/de OR 'abdominal surgery'/de OR 'abdominal wall closure'/de OR laparotomy/exp OR 'abdominal wall'/de OR (((incision* OR cicatri* OR scar* OR ventral*) NEAR/3 (herni*)) OR ((abdominal* OR transabdominal*) NEAR/3 (surger* OR clos* OR defect* OR wall*)) OR laparotom* OR (midline NEAR/3 incision*)):ab,ti) NOT ([animals]/lim NOT [humans]/lim)

### Medline (ovid) 490

("surgical mesh"/OR (mesh* OR 4DDOME OR AIGISRx OR AlloDerm OR AlloMax OR "Bard Composix EX" OR "BIO-A Tissue Reinforcement prosthesis" OR CollaMend OR DermaMatrix OR DualMesh OR "Evolution P3EM" OR FasLata OR FlexHD OR FortaGen OR "IntePro Lite" OR InteXen OR NEOVEIL OR "Parietex composite" OR Pelvicol OR Pelvisoft OR Pelvitex OR PerFix OR "Peri-Strips Dry" OR PeriGuard OR Permacol OR Physiomesh OR SeamGuard OR Strattice OR Surgisis OR "TiLoop Bra" OR Timesh OR Tutomesh OR Tutopatch OR Ultrapro OR Ventralex OR Veritas OR Vivosorb OR Vypro OR X-Repair OR XenMatrix).ab,ti.) AND ("Primary Prevention"/OR "prevention and control".xs. OR (prevent* OR protect* OR prophyla*).ab,ti.) AND ("Hernia, Ventral"/OR "Hernia, Abdominal"/OR abdomen/su OR laparotomy/OR "abdominal wall"/OR (((incision* OR cicatri* OR scar* OR ventral*) ADJ3 (herni*)) OR ((abdominal* OR transabdominal*) ADJ3 (surger* OR clos* OR defect* OR wall*)) OR laparotom* OR (midline ADJ3 incision*)).ab,ti.) NOT (exp animals/NOT humans/)

### Cochrane 30

((mesh* OR 4DDOME OR AIGISRx OR AlloDerm OR AlloMax OR 'Bard Composix EX' OR 'BIO-A Tissue Reinforcement prosthesis' OR CollaMend OR DermaMatrix OR DualMesh OR 'Evolution P3EM' OR FasLata OR FlexHD OR FortaGen OR 'IntePro Lite' OR InteXen OR NEOVEIL OR 'Parietex composite' OR Pelvicol OR Pelvisoft OR Pelvitex OR PerFix OR 'Peri-Strips Dry' OR PeriGuard OR Permacol OR Physiomesh OR SeamGuard OR Strattice OR Surgisis OR 'TiLoop Bra' OR Timesh OR Tutomesh OR Tutopatch OR Ultrapro OR Ventralex OR Veritas OR Vivosorb OR Vypro OR X-Repair OR XenMatrix):ab,ti) AND ((prevent* OR protect* OR prophyla*):ab,ti) AND ((((incision* OR cicatri* OR scar* OR ventral*) NEAR/3 (herni*)) OR ((abdominal* OR transabdominal*) NEAR/3 (surger* OR clos* OR defect* OR wall*)) OR laparotom* OR (midline NEAR/3 incision*)):ab,ti)

### Web-of-science 474

TS = (((mesh* OR 4DDOME OR AIGISRx OR AlloDerm OR AlloMax OR "Bard Composix EX" OR "BIO-A Tissue Reinforcement prosthesis" OR CollaMend OR DermaMatrix OR DualMesh OR "Evolution P3EM" OR FasLata OR FlexHD OR FortaGen OR "IntePro Lite" OR InteXen OR NEOVEIL OR "Parietex composite" OR Pelvicol OR Pelvisoft OR Pelvitex OR PerFix OR "Peri-Strips Dry" OR PeriGuard OR Permacol OR Physiomesh OR SeamGuard OR Strattice OR Surgisis OR "TiLoop Bra" OR Timesh OR Tutomesh OR Tutopatch OR Ultrapro OR Ventralex OR Veritas OR Vivosorb OR Vypro OR X-Repair OR XenMatrix)) AND ((prevent* OR protect* OR prophyla*)) AND ((((incision* OR cicatri* OR scar* OR ventral*) NEAR/3 (herni*)) OR ((abdominal* OR transabdominal*) NEAR/3 (surger* OR clos* OR defect* OR wall*)) OR laparotom* OR (midline NEAR/3 incision*))) NOT ((animal* OR rat OR rats OR mouse OR mice OR murine OR rabbit* OR rodent* OR pig OR sus OR swine* OR porcine OR monkey* OR dog OR sheep OR ovine) NOT (human* OR patient*)))

### Scopus 697

TITLE-ABS-KEY(((mesh* OR 4DDOME OR AIGISRx OR AlloDerm OR AlloMax OR "Bard Composix EX" OR "BIO-A Tissue Reinforcement prosthesis" OR CollaMend OR DermaMatrix OR DualMesh OR "Evolution P3EM" OR FasLata OR FlexHD OR FortaGen OR "IntePro Lite" OR InteXen OR NEOVEIL OR "Parietex composite" OR Pelvicol OR Pelvisoft OR Pelvitex OR PerFix OR "Peri-Strips Dry" OR PeriGuard OR Permacol OR Physiomesh OR SeamGuard OR Strattice OR Surgisis OR "TiLoop Bra" OR Timesh OR Tutomesh OR Tutopatch OR Ultrapro OR Ventralex OR Veritas OR Vivosorb OR Vypro OR X-Repair OR XenMatrix)) AND ((prevent* OR protect* OR prophyla*)) AND ((((incision* OR cicatri* OR scar* OR ventral*) W/3 (herni*)) OR ((abdominal* OR transabdominal*) W/3 (surger* OR clos* OR defect* OR wall*)) OR laparotom* OR (midline W/3 incision*))) AND NOT ((animal* OR rat OR rats OR mouse OR mice OR murine OR rabbit* OR rodent* OR pig OR sus OR swine* OR porcine OR monkey* OR dog OR sheep OR ovine) AND NOT (human* OR patient*)))

### cinahl (ebsco) 23

(MH "surgical mesh + " OR (mesh* OR 4DDOME OR AIGISRx OR AlloDerm OR AlloMax OR "Bard Composix EX" OR "BIO-A Tissue Reinforcement prosthesis" OR CollaMend OR DermaMatrix OR DualMesh OR "Evolution P3EM" OR FasLata OR FlexHD OR FortaGen OR "IntePro Lite" OR InteXen OR NEOVEIL OR "Parietex composite" OR Pelvicol OR Pelvisoft OR Pelvitex OR PerFix OR "Peri-Strips Dry" OR PeriGuard OR Permacol OR Physiomesh OR SeamGuard OR Strattice OR Surgisis OR "TiLoop Bra" OR Timesh OR Tutomesh OR Tutopatch OR Ultrapro OR Ventralex OR Veritas OR Vivosorb OR Vypro OR X-Repair OR XenMatrix)) AND (MH "Preventive Health Care" OR MW prevention OR (prevent* OR protect* OR prophyla*)) AND (MH "Hernia, Abdominal" OR MH abdomen/su OR MH laparotomy OR (((incision* OR cicatri* OR scar* OR ventral*) N3 (herni*)) OR ((abdominal* OR transabdominal*) N3 (surger* OR clos* OR defect* OR wall*)) OR laparotom* OR (midline N3 incision*))) NOT (MH animals + NOT humans +)

### Pubmed publisher 14

("surgical mesh"[mh] OR (mesh*[tiab] OR 4DDOME OR AIGISRx OR AlloDerm OR AlloMax OR "Bard Composix EX" OR "BIO-A Tissue Reinforcement prosthesis" OR CollaMend OR DermaMatrix OR DualMesh OR "Evolution P3EM" OR FasLata OR FlexHD OR FortaGen OR "IntePro Lite" OR InteXen OR NEOVEIL OR "Parietex composite" OR Pelvicol OR Pelvisoft OR Pelvitex OR PerFix OR "Peri-Strips Dry" OR PeriGuard OR Permacol OR Physiomesh OR SeamGuard OR Strattice OR Surgisis OR "TiLoop Bra" OR Timesh OR Tutomesh OR Tutopatch OR Ultrapro OR Ventralex OR Veritas OR Vivosorb OR Vypro OR X-Repair OR XenMatrix)) AND ("Primary Prevention"[mh] OR "prevention and control"[sh] OR (prevent*[tiab] OR protect*[tiab] OR prophyla*[tiab])) AND ("Hernia, Ventral"[mh] OR "Hernia, Abdominal"[mh] OR abdomen/su[mh] OR laparotomy[mh] OR "abdominal wall"[mh] OR (((incision*[tiab] OR cicatri*[tiab] OR scar*[tiab] OR ventral*[tiab]) AND (herni*[tiab])) OR ((abdominal*[tiab] OR transabdominal*[tiab]) AND (surger*[tiab] OR clos*[tiab] OR defect*[tiab] OR wall*[tiab])) OR laparotom*[tiab] OR (midline AND incision*[tiab]))) NOT (animals[mh] NOT humans[mh]) AND publisher[sb]

### Google scholar

Mesh|meshes prevention|preventive|protective|protection|prophylactic|prophylaxis "incisioal|cicatrical|scar|ventral hernia"|"abdominal|transabdominal surgery|closure|defect|wall"|laparotomy|"midline incision" -animal -animals -rats -mice

### Scielo 8

(Mesh*) AND (prevent* OR protect* OR prophyla*) AND ("incisional hernia" OR "cicatrical hernia" OR "scar hernia" OR " ventral hernia" OR "abdominal hernia" OR "abdominal surgery" OR "abdominal closure" OR "abdominal defect" OR "abdominal wall" OR laparotom* OR "midline incision")

### ScienceDirect 92

(Mesh*) AND (prevent* OR protect* OR prophyla*) AND ("incisional hernia" OR "cicatrical hernia" OR "scar hernia" OR " ventral hernia" OR "abdominal hernia" OR "abdominal surgery" OR "abdominal closure" OR "abdominal defect" OR "abdominal wall" OR laparotom* OR "midline incision") AND TOPIC (incisional hernia)

### ProQuest 9

(ti(Mesh*) OR ab(Mesh*)) AND (ti(prevent* OR protect* OR prophyla*) OR ab(prevent* OR protect* OR prophyla*)) AND (ti("incisional hernia" OR "cicatrical hernia" OR "scar hernia" OR " ventral hernia" OR "abdominal hernia" OR "abdominal surgery" OR "abdominal closure" OR "abdominal defect" OR "abdominal wall" OR laparotom* OR "midline incision") OR ab("incisional hernia" OR "cicatrical hernia" OR "scar hernia" OR " ventral hernia" OR "abdominal hernia" OR "abdominal surgery" OR "abdominal closure" OR "abdominal defect" OR "abdominal wall" OR laparotom* OR "midline incision"))
